# Effects of endometriosis on immunity and mucosal microbial community dynamics in female olive baboons

**DOI:** 10.1038/s41598-022-05499-y

**Published:** 2022-01-31

**Authors:** Nhung Le, Melissa Cregger, Asgerally Fazleabas, Andrea Braundmeier-Fleming

**Affiliations:** 1 Department of Obstetrics and Gynecology, Department of Medical Microbiology, Immunology and Cell Biology, Southern Illinois University School of Medicine, Springfield, IL 62702 USA; 2 Oak Ridge National Laboratory, Oak Ridge, TN USA; 3 Michigan State University, Grand Rapids, MI USA

**Keywords:** Immunology, Microbiology, Reproductive disorders

## Abstract

Endometriosis is defined as the growth of endometrial tissue in ectopic locations, and is associated with altered immune and microbial phenotypes. It is unclear if these changes are the result of the disease or may be causative. We induced endometriosis in non-human primates (*Papio Anubis*) to test *our hypothesis* that the growth of endometriotic lesions results in alterations in immune and microbial dynamics that may advance disease progression. Baboon samples were collected pre-inoculation (prior to disease induction), at 3, 6, 9, and 15 months after disease induction. Tolerant regulatory T-cells (Tregs) and inflammatory T-helper 17 (Th17) cells were identified in peripheral blood and within the eutopic/ectopic endometrial tissues. Microbiome communities were identified in fecal/urine samples. The induction of endometriosis decreased peripheral Tregs cells while Th17 cells increased at all post-induction collections, thus reducing the Tregs:Th17 cells ratio, indicating systemic inflammation. Microbiome diversity and abundance were altered at each sample site after disease induction. Thus, induction of endometriosis in baboons caused an immune shift toward an inflammatory profile and altered mucosal microbial profiles, which may drive inflammation through production of inflammatory mediators. Immune and microbial profiling may lead to innovative diagnostic tools and novel therapies for endometriosis treatment.

## Introduction

Endometriosis is defined as the presence of endometrial glands and stroma outside of the uterine cavity. In the US, endometriosis occurs in 1 out of every 10 women of reproductive age (over 200 million women worldwide^1,2^), and 45% of women with disease report sub-fertility or infertility^3^. Despite surgical and hormonal treatment of endometriosis, which can have initial positive response rates as high as 70%^4^, most women experience symptom recurrence requiring additional surgical intervention within 2 years^5^. Although endometriosis was first characterized in 1920’s, our understanding of the pathogenesis and pathophysiology of endometriosis remains an enigma, due to numerous reasons: (1) diagnosis of disease can take more than 10 years^6^, (2) variability in disease presentation and (3) lack of suitable animal models to investigate disease pathophysiology. In this study, we utilized the baboon *(Papio anubis*) model of induced experimental endometriosis to investigate physiological changes which occur at disease onset and throughout disease progression. The baboon model for endometriosis has been developed since the early 2000s^7^, and has several advantages over other animal models of the disease. These are (1) naturally menstruating primates, (2) demonstrate changes in the eutopic endometrium during the window of uterine receptivity, (3) develop spontaneous endometriosis similar to humans ^8^ and (4) adequate animal body size which allows for repetitive sample collection and sufficient sample size.

Endometriosis is a reproductive immune disorder where ectopic endometrial lesions display altered inflammatory profiles compared to a normal endometrium in humans and animal models^9,10^. Immune tolerant regulatory T cells (Tregs), including natural Tregs (nTregs; CD4^+^CD25^+^Foxp3^+^) and inducible Tregs (iTregs; CD4^+^CD25^−^Foxp3^+^), are prominent immune populations within reproductive tissues. Tregs are characterized by their expression of the Forkhead transcription factor p3 (Foxp3)^11,12^ and through the production of anti-inflammatory cytokines, IL-10 and TGFβ, which inhibit activation of T helper cells (Th). Inflammatory Th17 cells are derived from the CD4 lineage of T cells that secrete interleukin-17 (IL17s), IL21, IL22 and express the RAR-related orphan receptor gamma transcription factor (RORγt), to activate cytotoxic CD8^+^ T cells. Plasma and peritoneal fluid levels of IL-17 are elevated in endometriosis patients and we, along with others, have reported that Tregs and Th17 immune cell populations contribute to the systemic and tissue specific inflammatory profile of patients with endometriosis^13,14^. Briefly we found low levels of peripheral Tregs, greater Th17 cell populations and an increase in the Th17/Treg ratio, indicative of systemic inflammation^14^. We also reported that Tregs and Th17 localizations were enhanced within the ectopic endometrial implant, which promotes lesion development via induce angiogenesis^14^. Earlier studies using the baboon model demonstrated that the induction of endometriosis resulted in reduction of Tregs in the peripheral circulation and endometrium^12^. Together, these findings prompted us to characterize both Tregs and Th17 cell populations during the pathogenesis of endometriosis in the induced non-human primate model of disease.

Humans co-exist with resident microbiome that is composed of bacteria, viruses and fungi. The continuous and complex interactions between the mucosal surface and resident microbes shapes the host immunity. The mucosal immune homeostasis has not only protected the mucosal barrier surface against pathogens, it has also protects the commensal microbes against external insults^15,16^. The gastrointestinal (GI) microbiota plays an important role in the development of CD4^+^ T cells as evident by that germ free mice have smaller spleens and mesenteric lymph nodes and a decreased level of serum immunoglobulin^15,17^. The GI microbiota regulates the differentiation of Tregs and/or Th17 to induce a tolerant and/or inflammatory response toward a pathogenic or commensal-derived foreign antigen. For example, polysaccharide A molecules from *Bacteroides fragilis* can signal toll like receptor 2 (TLR2) on Tregs to subsequently suppress a Th17 response^18^ and segmented filamentous bacteria are potent inducers of Th17 cells^19,20^. The presence of specific bacterial species (*Lactobacillus*, *Bifidobacterium infantis*, etc*.*) in the urogenital (UG) tract protects mucosal epithelial cells by creating an unsuitable environment for pathogens to survive via the production of lactic acid which lowers the pH of the vaginal environment^21^. A shift in the composition of the GI/UG microbiota can cause either a pathological or protective outcome that is mediated by the regulation of Tregs and Th17 cells induced by the mucosal microbiota^22^. Therefore, it is reasonable to hypothesize that inflammation associated with endometriosis can shift the microbial dynamics of the GI/UG tracts and that these shifts may correlate with presence of the disease and the disease severity.

We hypothesize that induction of endometriosis in *baboons *(*P. Anubis*) *results in chronic systemic and tissue specific inflammation through regulation of Th17 and Treg populations. Further, the induction of endometriosis altered gastrointestinal/urogenital microbial communities that are distinct from non-diseased animals*. Utilizing this model, the aims of our study were to (1) identify the immune phenotype as well as microbial phenotypes in non-diseased (pre-inoculation) phase and throughout the progression of disease pathogenesis; and (2) to investigate the correlation of the immune phenotypes with microbial communities at each surgical collection time point to further understand how these dynamics shift after experimental induction of endometriosis. Overall, these investigations have allowed us to profile immune and microbial profiles at the onset of endometriosis and throughout disease progression.

## Results

### Peripheral Treg and Th17 cell populations were altered by induction of endometriosis

**Figure 1:**
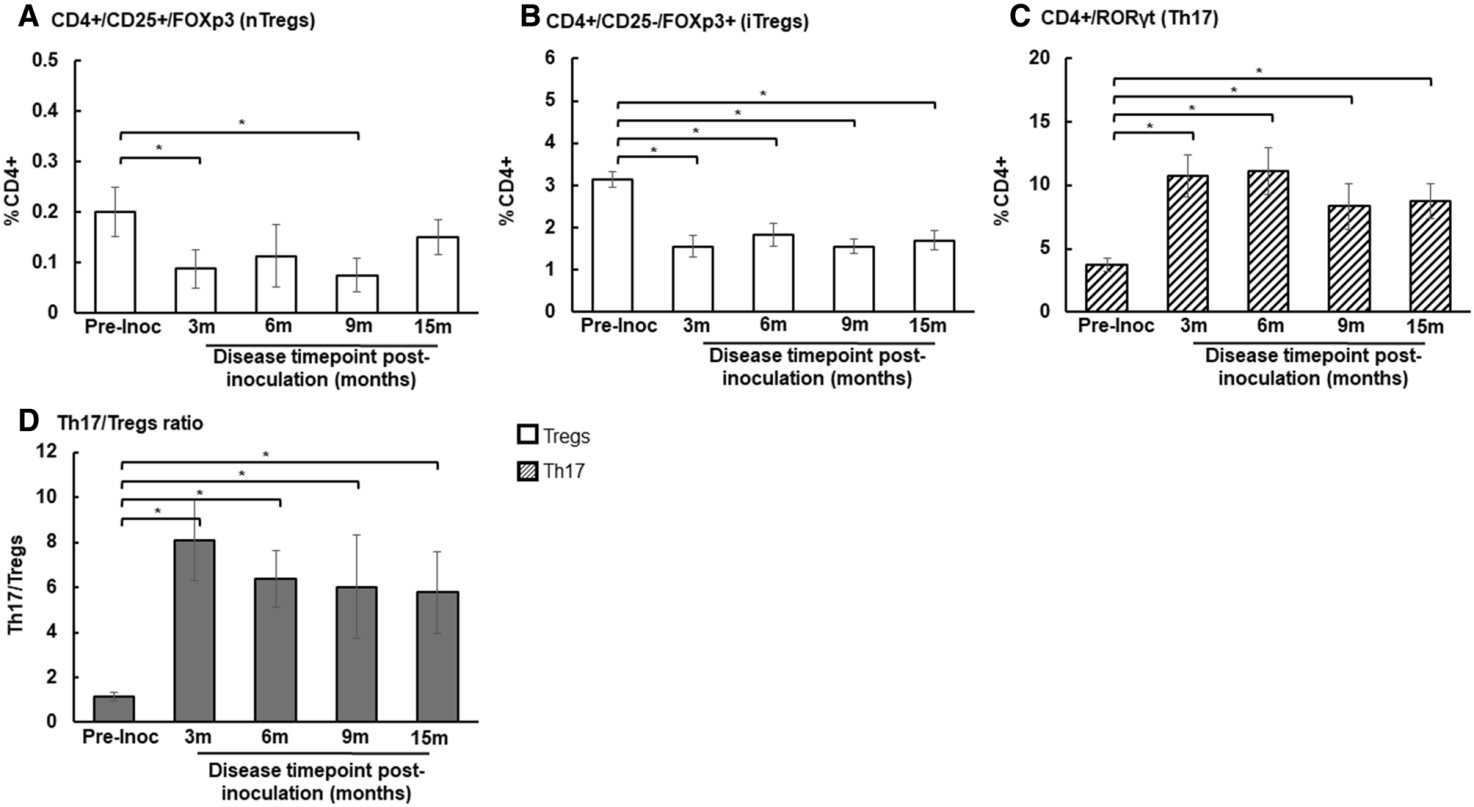
Immune cell population in peripheral blood samples of non-human primates. (**A–C**) Peripheral Treg and Th17 cell populations were measured for 8 baboons at pre-inoculation and post-inoculations: 3, 6, 9 and 15 months. (**A**) Natural Tregs (nTregs); (**B**) Inducible Tregs (iTregs); (**C**) Th17 population. (**D**) Th17/Tregs ratio. *Indicates significance between groups. Mann–Whitney *U*-test, *p*-value < 0.05.

To determine if induction of endometriosis altered peripheral immune cell populations, we identified nTregs (CD4^+^CD25^+^Foxp3^+^), iTregs (CD4^+^CD25^−^Foxp3^+^) and Th17 cell populations in blood samples collected over time (Fig. 1). We demonstrated that post induction, animals exhibited systemic inflammation through an alteration of tolerant and inflammatory T cell populations (Fig. 1).

Initially, the induction of disease significantly reduced peripheral nTregs at 3 months and 9 months post-inoculation (Fig. 1A). The iTregs cell population was reduced at 3 months post-inoculation and remained decreased at each following time point (Fig. 1B). Conversely, the peripheral Th17 cell population was increased at each post-inoculation timepoint (Fig. 1C). To determine if the induction of endometriosis altered peripheral immune homeostasis (i.e., balance between inflammation and tolerance), we analyzed the ratio of Th17 to Tregs (inducible + natural) cell populations. The ratio of Th17 to Tregs populations increased at 3 months post-inoculation and remained elevated at each following surgical time points, indicative of systemic inflammation (Fig. 1D). These results suggested that the induction of endometriosis altered both tolerant and inflammatory immune populations which disrupted immune homeostasis and this disruption was maintained as long as peritoneal endometriotic lesions were present.

### Foxp3 and RORγt in eutopic and ectopic endometrium of non-human primates

**Figure 2:**
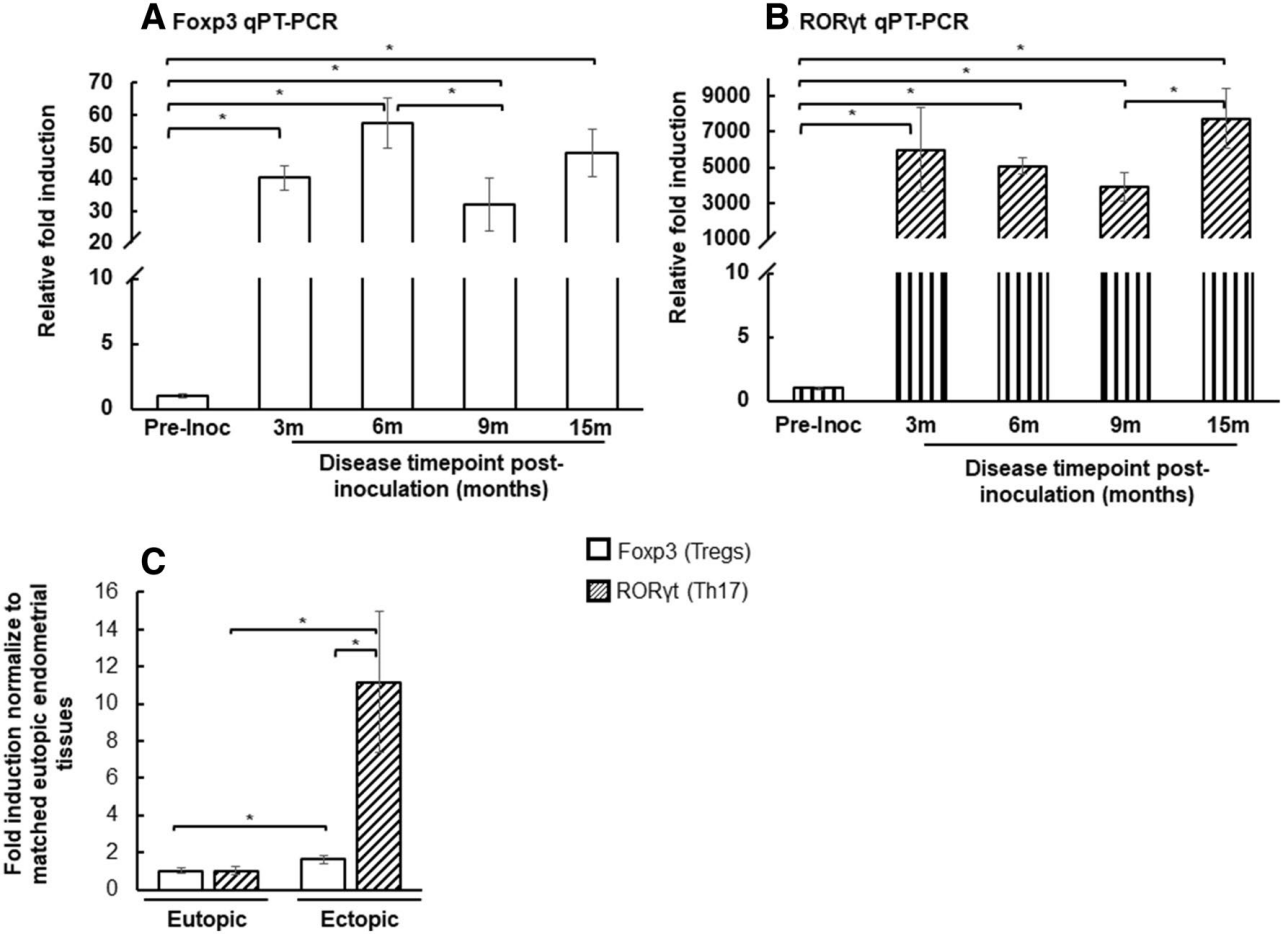
Foxp3 and RORγt quantitative RT–PCR of non-human primates eutopic and ectopic endometrial tissues. (**A**) Foxp3, and (**B**) RORγt transcript levels were measured in the eutopic endometrium tissues of 8 baboons at pre-inoculation: 3, 6, 9 and 15 months. The relative fold induction of Foxp3 and RORγt genes was normalized to H3.3 endogenous gene for all experimental conditions. *Indicates significant difference between compared groups. (**C**) Fold induction for each eutopic to matched ectopic endometrial tissues at 15 months collection. The ectopic endometrial tissues was normalized to matched eutopic endometrial tissues for all 8 animals. Mann–Whitney *U*-test, *p-*value < 0.05.

To investigate the effect of disease induction and disease progression on activation of Tregs and Th17 in eutopic endometrial tissues, we measured the expression of transcription factor RORγt (Th17) and Foxp3 (Tregs) in eutopic endometrial tissues collected at each surgical time point (Fig. 2). We observed an elevation of Foxp3 and RORγt transcripts levels in the eutopic endometrium at each time point after disease induction (Fig. 2A,B). Overall, within the eutopic endometrium the fold induction of RORγt transcripts were significantly higher than the Foxp3 transcripts following the induction of endometriosis; thus, driving an inflammatory profile in the eutopic endometrium, (Fig. 2A,B). At 15 months of the disease, we collected ectopic endometrial tissues which allowed us to compare RORγt and Foxp3 transcript expression in matched eutopic and ectopic endometrial tissues. RORγt and Foxp3 transcripts were elevated in ectopic endometrium compared to matched eutopic samples (Fig. 2B). These data indicated that the eutopic endometrium has enhanced expression of RORγt and Foxp3 transcripts throughout disease progression and that these expression patterns are even more augmented in ectopic endometrial tissues. Altogether, a lower number of Treg cells in the peripheral blood suggested an increase in systemic inflammation via removal of the inhibition of Th17 cell function by Tregs; but a higher number of Treg cells in the eutopic and ectopic endometrium allows ectopic endometrial tissues to attain immune tolerance from the innate immune system and may promote disease establishment.

### Bacterial community diversity following induction of endometriosis

**Figure 3:**
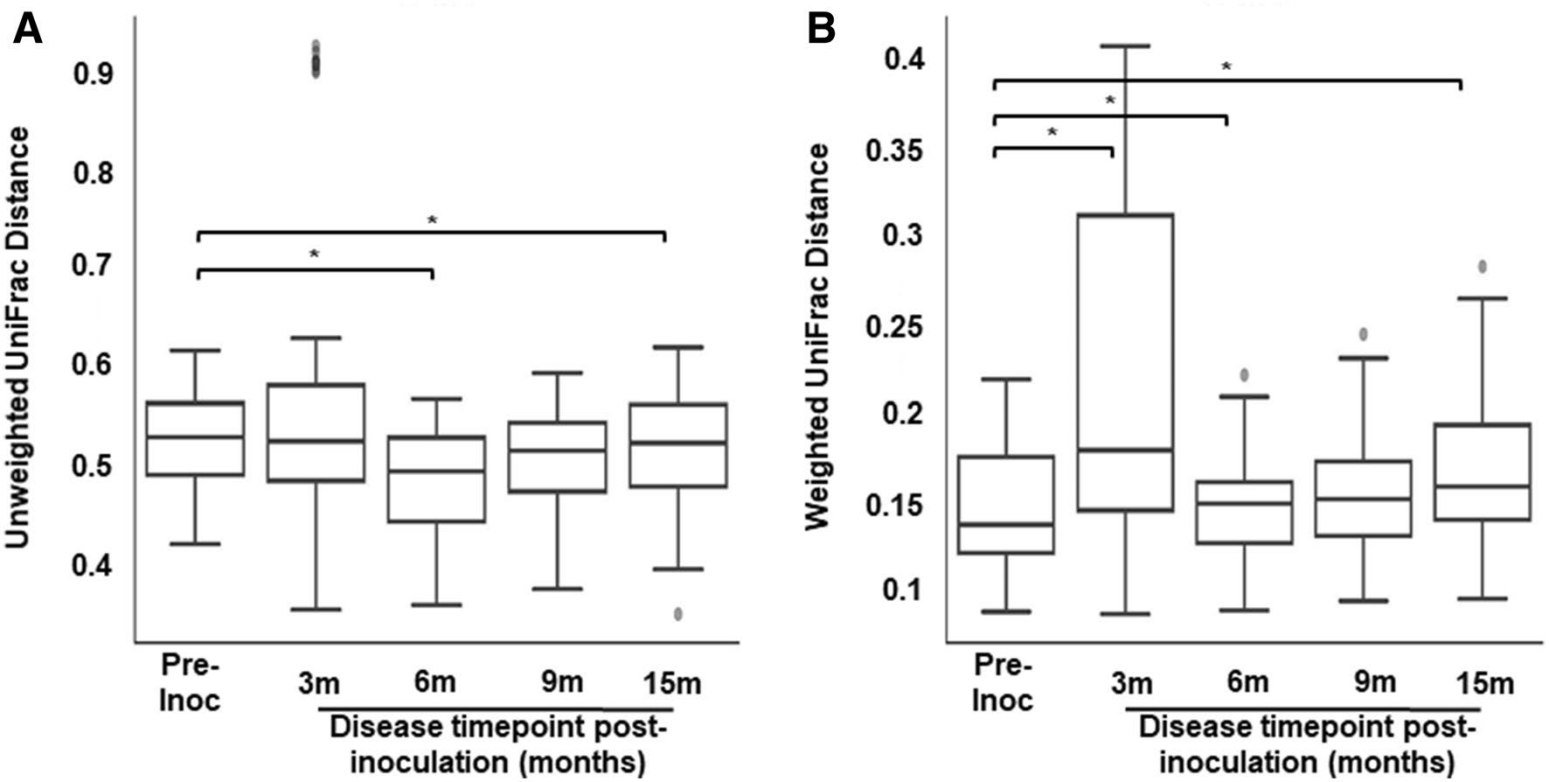
β diversity in gastrointestinal tract (GI) from pre- and post-inoculation of endometriosis using the ANOSIM algorithm. (**A**) Unweighted Unifrac, (**B**) Weighted Unifrac. The horizontal lines inside the boxes indicate the median, whereas the lower lines and upper lines of the boxes indicate the 25th and the 75th percentiles, respectively. The dots outside the boxes indicate the outliers. *Indicates significance between groups, *p-*value < 0.05. (Pre-inoc and 3 m post-inoc: unweighted p = 0.38, weighted p = 0.021; pre-inoc and 6 m post-inoc: unweighted p = 0.04, weighted p = 0.029; pre-inoc and 9 m post-inoc: unweighted p = 0.11, weighted p = 0.38; pre-inoc and 15 m post-inoc: unweighted p = 0.043, weighted p = 0.04).

To compare differences in microbial diversity over disease progression, among all animals by sample types, we performed beta-diversity analyses that used phylogenetic information with unweighted and weighted UniFrac distance metrics. The unweighted Unifrac (qualitative) measured the fraction of branch length in a phylogenetic tree that leads to descendants of one sample or the other while a weighted UniFrac (quantitative) directly accounted for differences in relative abundances of each type of organism. GI bacterial communities were significantly different between pre-inoculation and throughout the disease progression, except at 9 months post-inoculation (Fig. 3A,B). There was no changes in microbial diversity between study time points for the UG tract (vaginal swabs) or peritoneal cavity (peritoneal fluid) (Supplementary Fig. S1). Thus, the induction of endometriosis, in non-human primates, altered GI bacterial diversity as disease progressed but this change was not evident in UG or peritoneal bacterial communities.

### Bacterial composition alterations with the induction of endometriosis

**Figure 4:**
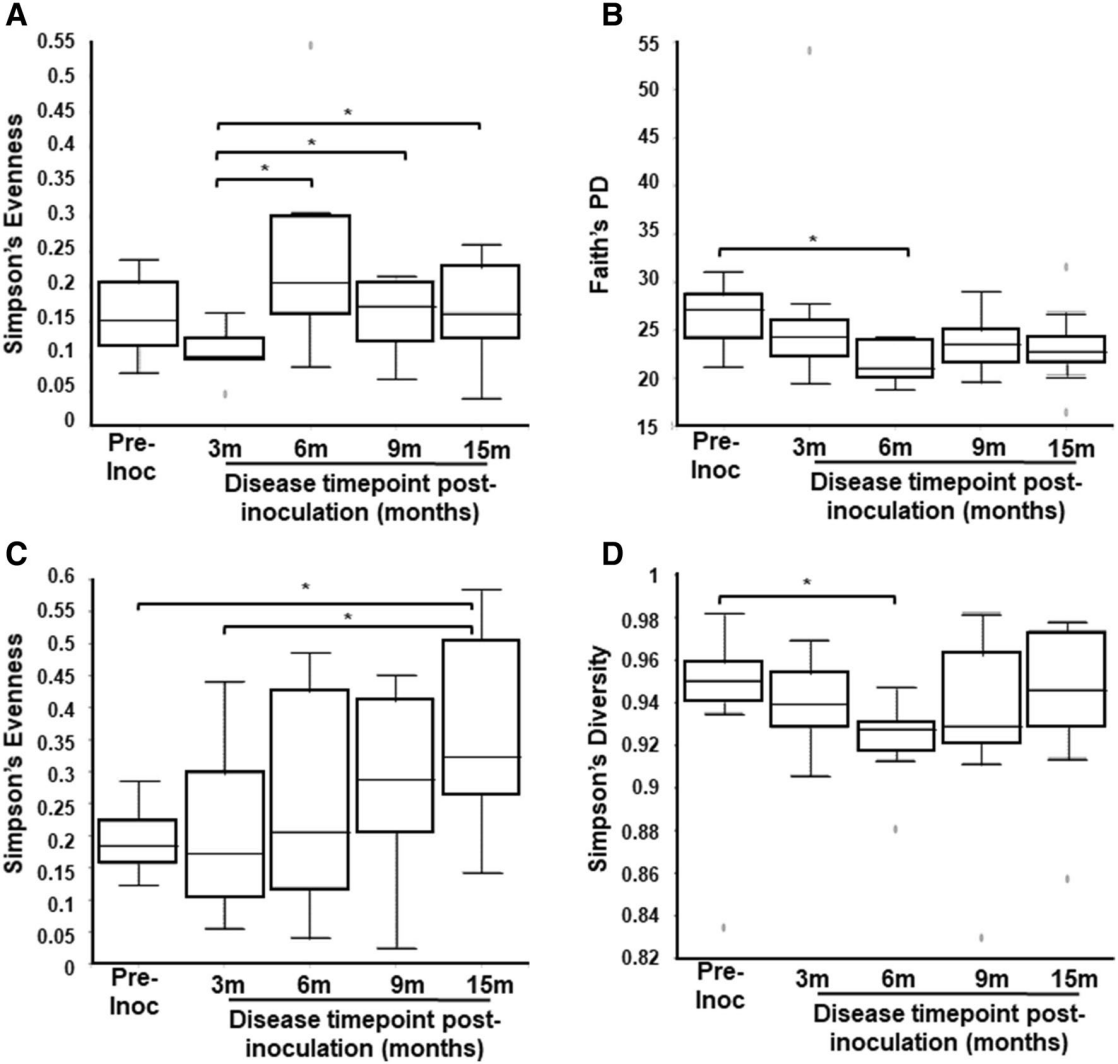
α diversity from pre- and post-inoculation of endometriosis in GI tract (**A**, **B**) and urine (**C**, **D**). (**A**): Simpson’s evenness in GI tract (Simpson’s evenness [species uniformity]: pre-inoc and 3 m post-inoc: p = 0.09; 3 m and 6 m post-inoc: p = 0.02; 3 m and 9 m post-inoc: p = 0.034; 3 m and 15 m post-inoc: p = 0.04), (**B**) Faith’s phylogenetic diversity in GI tract (p = 0.01), (**C**) Simpson’s evenness in urine (Simpson’s evenness: pre-inoc and 15 m post-inoc: p = 0.03; 3 m and 15 m post-inoc: p = 0.04), (**D**) Simpson’s diversity in urine (Simpson’s diversity: pre-inoc and 6 m post-inoc: p = 0.03). The horizontal lines inside the boxes indicate the median, whereas the lower lines and upper lines of the boxes indicate the 25th and the 75th percentiles, respectively. The dots outside the boxes indicate the outliers. *Indicates significance between groups, *p*-value < 0.05.

To assess if the induction of endometriosis caused differences in bacterial community compositions (species richness and uniformity), we performed alpha-diversity analysis with PERMANOVA for Simpson’s evenness, Simpson’s diversity and Faith’s phylogenetic diversity. Simpson’s evenness measures how evenly the abundance was distributed among species while Simpson’s diversity measured the number of species presented in a community. Additionally, Faith’s phylogenetic diversity incorporated phylogenetic difference between species via summing branch lengths on phylogenetic trees. Overall, GI bacterial evenness was reduced at 3 months post-inoculation, but recovered at 6 months to 15 months post-inoculation (Fig. 4A). Faith’s phylogenetic diversity was lower for all animals at 6 months post-inoculation compared to pre-inoculation (Fig. 4B). There was no difference in GI Simpson’s diversity (species richness) at each time point. Urinary bacterial alpha-diversity (Simpson’s evenness and Simpson’s diversity) was reduced at 3, 6 and 15 months post-inoculation (Fig. 4C,D). The vaginal tract and the peritoneal cavity bacterial alpha diversities did not alter during the disease induction and throughout the disease progression (Supplementary Table S1). Thus, the induction of endometriosis, in non-human primates, altered GI and urinary bacterial alpha-diversity.

### Taxonomic variation with induction of endometriosis

**Figure 5:**
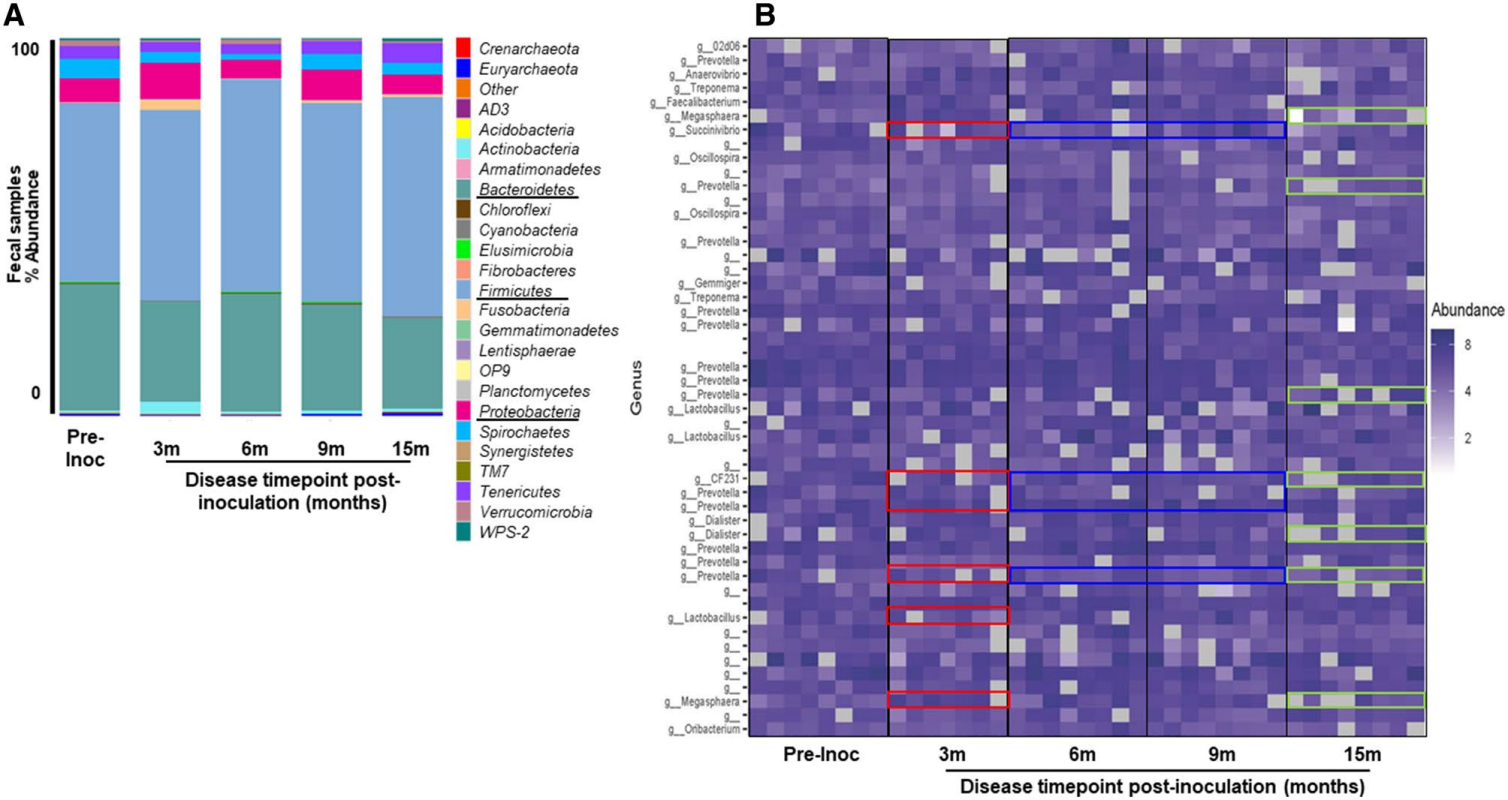
Taxonomical analysis for fecal samples of 8 non-human primates from pre- and post-inoculation of endometriosis. (**A**) Level 2 (phyla) taxonomical summary plots. (**B**) The top 50 abundant bacterial genera in GI tract. Samples were collected from animal at the pre-inoculation (pre-Inoc) (left panel) and following disease induction (right panel).

Induction of endometriosis altered mucosal microbiota in the GI/UG tracts. *Firmicute* species were abundant in the GI tract of all animals, regardless of disease status (Fig. 5A). The most abundant phylum identified from fecal samples was Firmicutes followed by Bacteroidetes and Proteobacteria (Fig. 5A, black underline). At the genus level, prior to disease induction, Prevotella dominated GI bacterial communities; this was followed by *Megasphaera*, *Lactobaccillus*, *Oscillospira*, *Anaerovibrio*, *02d06*, *Treponema*, *Succinivibrio* and *CF231* (Fig. 5B). Disease induction resulted in decreased levels of *Succinivibrio*, *Prevotella*, *Megasphaera*, *Lactobaccillus* and *CF231* at 3 months post-inoculation, but the levels of *Succinivibrio*, *Prevotella*, and *CF231* increased throughout disease progression from 6 to 9 months post-inoculation (Fig. 5B, blue box). However, as the disease progressed, levels of other genera such as *Megasphaera, Treponema* and *Prevotella* were also decreased (Fig. 5B, green box).

**Figure 6:**
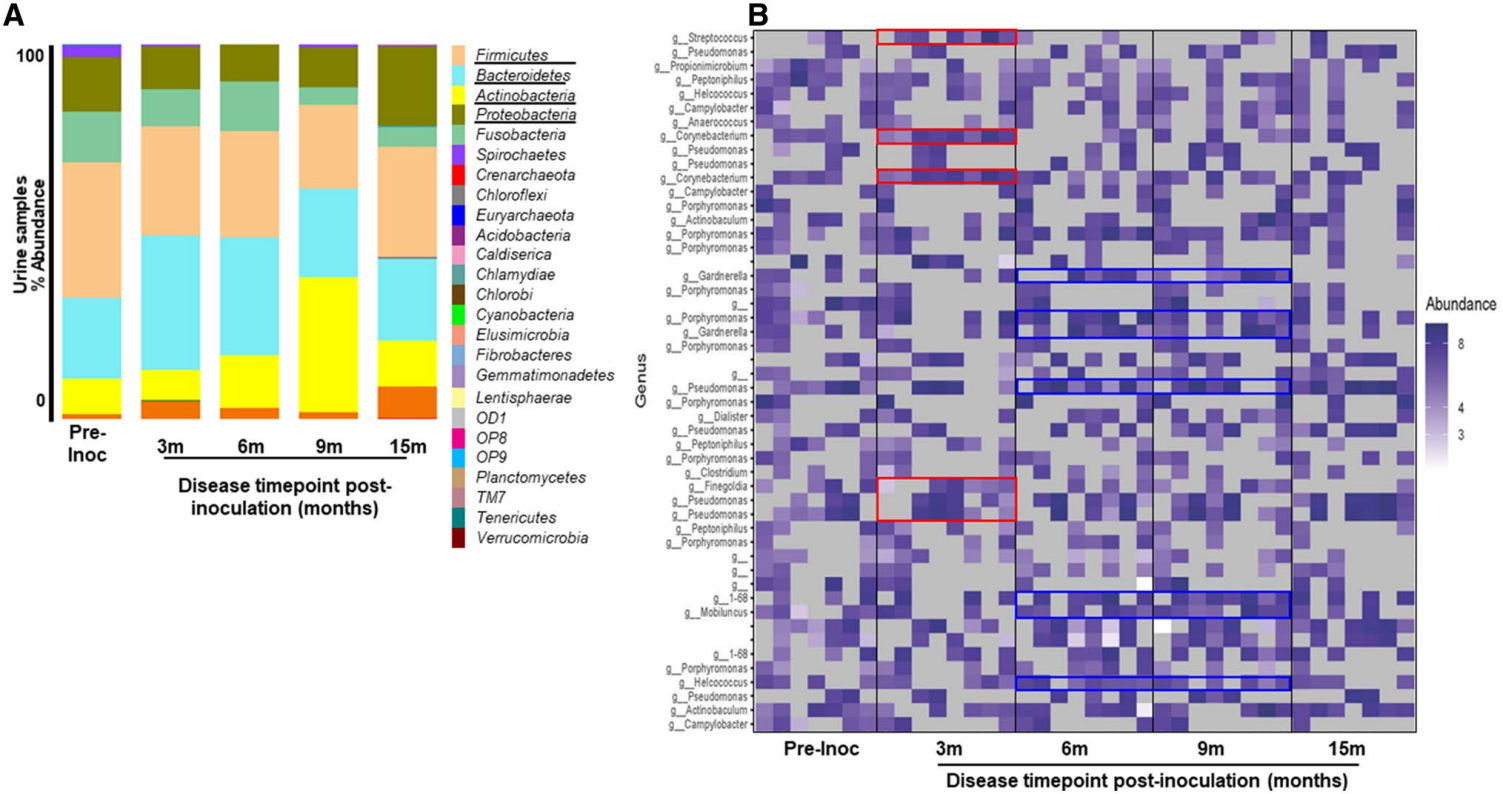
Taxonomical analysis for urine samples of 8 non-human primates from pre- and post-inoculation of endometriosis. (**A**) Level 2 (phyla) taxonomical summary plots. (**B**) The top 50 abundant bacterial genera in urinary tract. Samples were collected from animal at the pre-inoculation (pre-inoc) (left panel) and following disease induction (right panel).

Similar to fecal samples, disease induction shifted urinary microbial dynamics upon comparison of pre-inoculation samples to those collected at 3- to 15-months post-inoculation (Fig. 6A). The Firmicutes phylum was predominant followed by Bacteroidetes, Antinobacteria and Proteobacteria (Fig. 6B, black underline). Prior to the disease induction, urinary bacterial communities were dominated by the genera *Porphyromonas*, *Pseudomonas*, *Campylobacter*, *Corynebacterium*, *Acitinobaculum*, and *Streptococcus* (Fig. 6B). After the disease induction, the levels of *Corynebacterium, Pseudomonas,* and *Streptococcus* increased (Fig. 6B, red box), while other genera were decreased in these animals at 3 months post-inoculation (Fig. 6B). *Pseudomonas*, *Porphyromonas*, *Garnerella*, and *Helcococcus* were increased in the urinary tract at 6 months and 9 months post-inoculation (Fig. 6B, blue box). As the disease further progressed, the levels of multiple genera once again decreased in the urinary tract at 15 months post-inoculation (Fig. 6B, right panel).

In the vagina, Bacteroidetes was the predominant phylum, followed by Firmicutes, Antinobacteria, and Fusobacteria (Fig. S2). At the genus level, prior to disease induction, *Porphyromonas*, *Mobiluncus*, *Treponema*, *Campylobacter*, *Prevotella*, and* Streptobacillus* dominated vaginal bacterial communities. However, throughout the disease progression these genera within the vagina diminished and were never restored. We also observed a dominant increase of the phylum Firmicutes (*Peptoniphilus* and *Dialister*) after the disease induction.

For the peritoneal cavity, an unclassified group of bacteria was dominant in the peritoneal bacterial communities and proteobacteria was the next predominant phylum, followed by Firmicutes and Antinobacteria (Fig. S2). Prior to disease induction, the dominant genera were *Lactobacillus, 02d06, Campylobacter* and *Succinivibrio* but these diminished upon disease induction and did not restore during the disease development. We observed an increase of the phyla Firmicutes (*Phascolarctobacterium* and *Helcococcus*) and Proteobacteria (*Campylobacter*) in these animals upon disease induction.

Finally, we wanted to determine if induction of disease was caused the development of an inflammatory disorder. While typically only analyzed in gut, we investigated the ratio of Firmicute/Bacteroidetes within each sample type. The GI tract had the lowest ratio of Firmicute/Bacteroidetes pre-inoculation, but this gradually increased at each of the collection time point during the disease development (in GI tract: pre-inoculation = 1.34; post-inoculation: 3 m = 1.9; 6 m = 1.8; 9 m = 1.8; 15 m = 2.4). However, in urine, we observed a decrease in the ratio at each study time point after the induction of endometriosis (pre-inoculation = 1.68; post-inoculation: 3 m = 0.8; 6 m = 0.9; 9 m = 0.9; 15 m = 1.3). In the vagina and peritoneal cavity, the Firmicute/Bacteroidetes ratio fluctuated throughout the disease establishment and progression (vaginal tract: pre-inoculation = 0.6; post-inoculation: 3 m = 1.2; 6 m = 1.1; 9 m = 0.5; 15 m = 0.8; peritoneal cavity: pre-inoculation = 4; post-inoculation: 3 m = 5; 6 m = 1.8; 9 m = 4).

Taken all together, these results indicated that the induction of endometriosis altered the mucosal microbiota in multiple sites and additionally, the presence of endometriotic lesions altered the quantity and composition of microbial species. Disease establishment and progression further distinguished the microbiome profiles of these animals from the healthy control time point (pre-inoculation).

### Association of Immune populations with microbial dynamics

To expand upon our analysis of how microbial dynamics impacted immune status, we performed correlative analyses to determine if an alteration of microbial diversities via the induction of endometriosis was associated with peripheral immune cells populations (iTregs, nTregs and Th17) (Supplementary Table S2). The GI microbial alpha-diversity was positively correlated with circulating nTregs cells at 3 months post-inoculation (p = 0.008), while iTregs cell populations was associated with GI alpha-diversity at 9 months post-inoculation (p = 0.03). Urinary microbial alpha-diversity correlated with peripheral nTregs at 3 months and 15 months post-inoculation (p = 0.002; p = 0.02 respectively) and correlated with Th17 cell population at 6 months post-inoculation (p = 0.04). These results showed that the induction of disease altered bacterial diversity at both sites (GI/urine) moreover, changes in microbial diversity were associated with distinct sub-types of T cells at different stages of disease progression. Immune tolerant cells (Treg sub-types) were associated with microbial diversity during early and later stages of disease progression whereas inflammatory cells (Th17) was associated with microbial diversity in the middle of our timeline. These data suggest that not only are the microbial and immune profiles transient but there exists a dynamic relationship between microbial and immune parameters during disease progression.

### Association between microbial species and peripheral immune cells with induction of endometriosis

**Figure 7:**
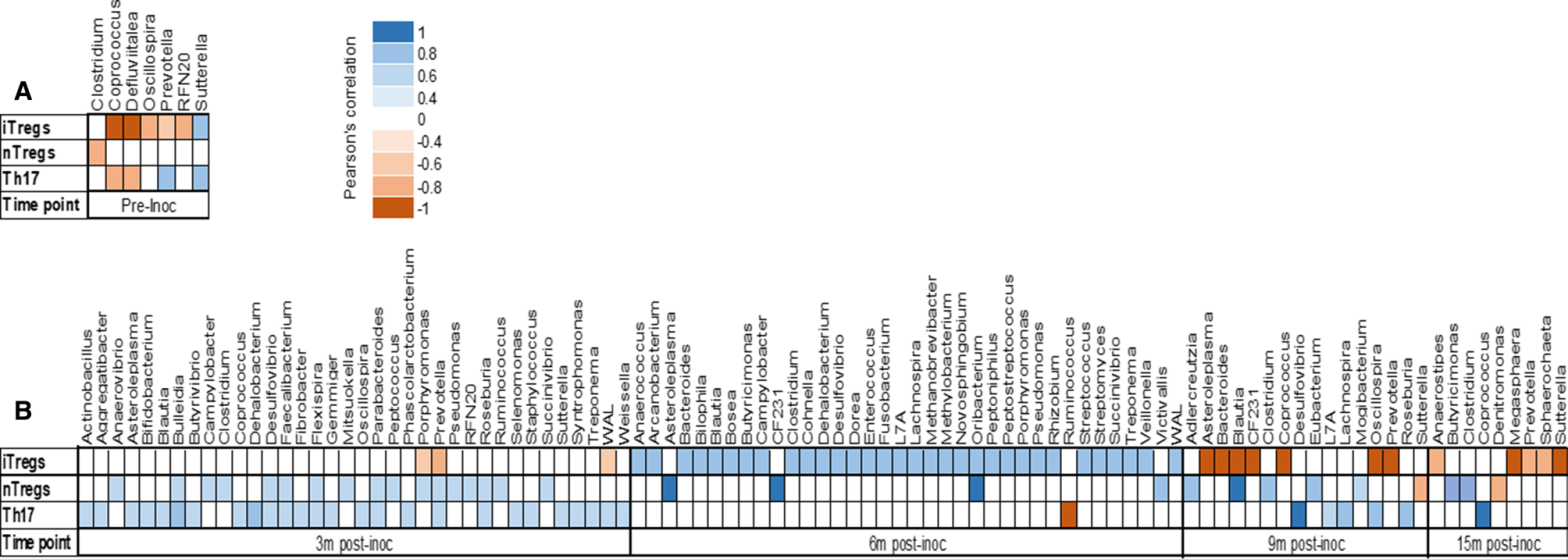
Pearson’s correlation coefficient of GI bacterial communities with level of peripheral immune cell populations (iTregs, nTregs, Th17) in 8 non-human primates from pre- and post-inoculation of endometriosis. (**A**) Pre-inoculation. (**B**) Post-inoculation.

To examine if bacterial community structure was associated with immune phenotypes, we performed Pearson’s correlation coefficient for each bacterial site with the peripheral iTregs, nTregs and Th17 cell populations. In the GI tract, prior to the induction of endometriosis, the phyla Bacteroidetes, Firmicutes, and Proteobacteria (Genera: *Clostridium*, *Coprococcus*, *Defluviitalea*, *Oscillospira*, *Prevotella*, *RFN20*) were negatively correlated with level of peripheral nTregs and iTregs cells; meanwhile *Prevotella* and *Sutterella* were positively correlated with level of Th17 cell populations (Fig. 7A). At 3 months post-inoculation, there were additional phyla (Actinobacteria, Euryarchaeota, Fusobacteria, Lentisphaerae, Spirochaetes, and Synergistetes) in GI communities that had a positive correlation with peripheral nTregs and Th17 cell populations; only *Porphyromonas, Prevotella,* and *WAL* were negatively correlated with the level of iTregs cells (Fig. 7B, left panel). At 6 months post-inoculation, GI bacteria positively correlated with the level of iTregs cell population, whereas a lower amount of GI species was positively correlated with peripheral nTregs cells (Fig. 7B, middle panel). At 9 months and 15 months post-inoculation, we detected a higher number of GI bacteria that negatively correlated with the level of iTregs cells compared to 3 months and 6 months post-inoculation (Fig. 7B, right panel). Overall, the induction of endometriosis resulted in a higher number of GI bacteria that correlated with immune cell populations (nTregs, Th17) at 3 months and 6 months post-inoculation; meanwhile at 9 months and 15 months post-inoculation, there was a reduction in the number of GI species that correlated with these immune cell populations (Fig. 7B).

**Figure 8:**
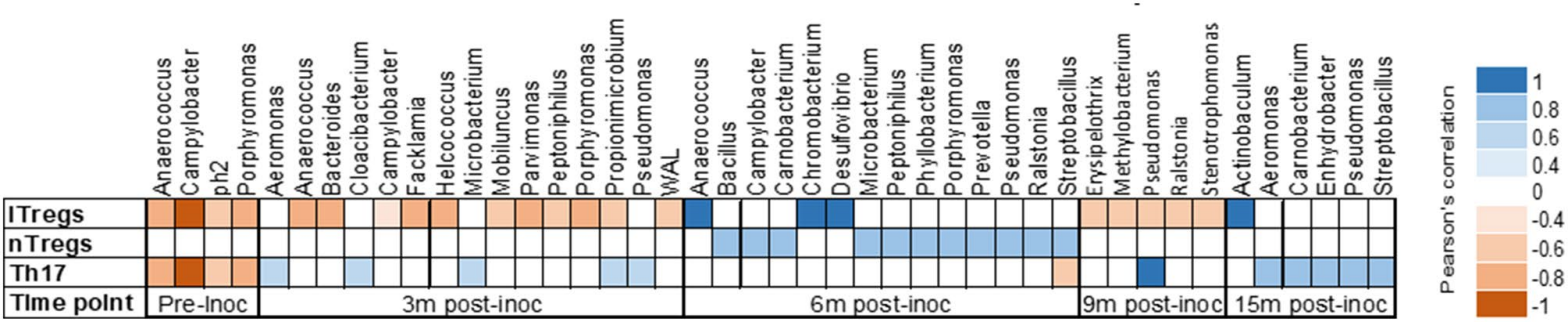
Pearson’s correlation coefficient of urinary bacterial communities with level of peripheral immune cell populations (iTregs, nTregs, Th17) in 8 non-human primates from pre- and post-inoculation of endometriosis.

In urine, at pre-inoculation, the phyla Actinobacteria, Bacteroidetes, Firmicutes, and Proteobacteria were negatively correlated with the levels of iTregs and Th17 cell populations (Fig. 8, left panel). At 3 months post-inoculation, urinary bacteria were negatively correlated with the peripheral iTregs cell population but were positively correlated with the level of Th17 cells (Fig. 8, middle panel). No correlation was noted between urinary species and circulating nTregs cells at pre-inoculation and at 3 months post-inoculation (Fig. 8). At 6 months post-inoculation, there were positive correlations between the urinary bacteria and levels of peripheral iTregs and nTregs cells, while only *Straptobacillus* was negatively correlated with circulating Th17 cell population (Fig. 8, middle panel). A negative correlation was identified for urinary species and the peripheral iTregs cell population at 9 months post-inoculation. No correlation was detected between urinary species and the circulating nTregs cells at 9 months and at 15 months post-inoculation (Fig. 8). Finally, at 15 months post-inoculation, there was a positive correlation for urinary bacteria and the level of Th17 cell population (Fig. 8). We did not detect any correlation between immune populations and vaginal or peritoneal samples (Supplementary Table S3).

In summary, the induction of peritoneal endometriosis altered bacterial communities within the GI and UG tracts of animals with endometriosis. Specifically, the dysbiosis of GI/UG microbial communities was associated with aberrant levels of peripheral nTregs, iTregs (immune tolerant), and Th17 (inflammatory) cell populations.

## Discussion

The immune system and the commensal bacterial species in the gut and the female reproductive tract play a major role in preserving overall homeostasis for the host’s health. These microbiota support immunological regulation of reproductive functions, and are influenced by factors such as immunological responses, metabolic changes and the environment^23^. Therefore, a shift in the commensal microbial communities are indicative of potential shifts in immune signaling and function. Utilizing a non-human primate animal model of induced endometriosis in olive baboons, this study investigated the alteration of immune populations and microbial dynamics in response to establishment of disease.

*Our first goal was to define the endometriosis-associated inflammation throughout the progression of endometriosis by characterizing Tregs and Th17 profiles using the non-human primate induced model of endometriosis.* Increased Th17 cells and their cytokine profiles have been observed in the peritoneal fluid of women with endometriosis^24^, and excessive IL-17 from Th17 cells is associated with the severity of disease^25^. However, there are limited publications regarding the levels of Th17 cells in animal models of endometriosis. Consistent with previous reports from human studies, Th17 populations in baboons were expressed most abundantly in the peripheral circulation throughout the disease pathogenesis^12,26,27^. Similar to previous reports from Braundmeier et al., the induction of endometriosis resulted in a rapid decrease in both nTregs and iTregs in the peripheral circulation. Thus, our data demonstrates a systemic immune imbalance (enhanced Th17/Treg ratio) after the induction of endometriosis; furthermore, this immune phenotype persists throughout the progression of the disease (15 months). In addition, we observed an upregulation of both Foxp3 and RORγt transcripts in the eutopic and matched ectopic endometrial tissues, which supports similar reports in human studies^28^. The immune system’s failure to down regulate Foxp3 expression in the eutopic endometrial tissues during the disease may enhance ectopic endometrial growth through inhibition of immune clearance^29^. Alternatively, the higher expression of Th17 and lower Treg transcript within endometriotic lesions may also stimulate the continuous RORγt cell proliferation in endometriosis, which leads to the infiltration of inflammatory cells and mediators into the lesion(s), thus promoting tissue remodeling.

*Our second goal was to define microbial composition after the induction of endometriosis and identify the potential variations associated with disease.* We started by assessing the bacterial species richness and diversity at pre-inoculation and throughout the progression of endometriosis. The GI microbiome diversity is crucial in health maintenance as microbiota and their metabolites (short chain fatty acids [SCFAs] and microbially transformed bile acids) have been proven to play a fundamental role in immune cell regulation and signaling^30,31^. In this regard, the gut microbial communities (e.g. *Clostridia* spp.) serve as a source of SCFAs such as butyrate and propionic acid, which help to maintain Tregs cell expansion (immunosuppressive function) and promote an intestinal homeostasis^30,31^. The microbiota within the UG tract is dominated by *Lactobacillus* species. These commensal bacteria in the UG tract create an unsuitable environment for pathogens and keep them from colonizing and causing infection by producing lactic acid as a fermentation byproduct that lowers the pH of the UG tract environment (pH < 4.5). A reduction in microbial diversity due to dysbiosis and inflammation reduces their metabolic activity, which might alter immune homeostasis. We observed that the GI bacterial communities were altered in their diversity and richness after the induction of the disease but recovered later as the disease progressed (6 months to 15 months post-inoculation). Urinary bacterial diversity was reduced after the induction of endometriosis and remained altered throughout disease progression. These results showed that induction of the disease altered bacterial diversity at both sites (GI/urogenital tracts), but unlike the urogenital tract the GI microbial dynamics were transient in the response to disease induction.

Consistent with previous studies, more than 80% of all study animals’ microbiota were composed of *Actinobacteria*, *Bacteroidetes*, *Firmicutes*, and* Proteobacteria*^16,32,33^. An elevated ratio of *Firmicutes/Bacteroidetes* has been associated with obesity^34,35^, colorectal cancer^36^, and rheumatoid arthritis^37^. A recent review from Magne et al., suggested that using *Firmicutes/Bacteroidetes* ratio to determine health status would be a challenge due to multiple discrepancies such as lifestyle associated factors and the sampling process. In this study, the ratio of *Firmicute/Bacteroidetes* in the GI tract gradually increased at each of the collection timepoints during disease development. However, in the urinary tract, we observed a decrease in the ratio of *Firmicute/Bacteroidetes* at each study timepoint after the induction of endometriosis. The reduction in the levels of *Succinivibrio, Megasphaera* and *Prevotella* spp. (phylum of *Proteobacteria*, *Firmicutes*, and *Bacteroidetes* respectively) observed after the induction of endometriosis may play a role in endometriosis-associated inflammation at 3 months post-inoculation. Indeed, *Prevotella* spp. are known to stimulate the production of anti-inflammatory cytokines, such as IL-10, using Foxp3^+^ regulatory T cells through the production of propionate, succinate and acetate during the carbohydrate fermentation of organic acids^38^. The level of propionic acid increases during infection to reduce inflammation and protects tissues during the immune response to an infection^38–40^. Similarly, high levels of *Clostridium* spp. (*Firmicutes* phylum) induce colonic Foxp3^+^ regulatory T cells and activate T cell dependent immunoglobulin A production^38,41,42^. Based on a comparison between pre-inoculation samples to those collected at 3- to 15-months post-inoculation, disease induction increased the pathogenic bacteria (e.g. *Corynebacterium*, *Pseudomonas*, and *Streptococcus*) in urine. Additionally, the presence of endometriotic lesions altered both quantity and microbial species composition.

Additionally, we investigated the association between the GI and urinary microbiome and the peripheral circulating immune cells in non-human primates with endometriosis. Overall, disease induction resulted in more GI bacteria that were positively correlated with immune cell populations (nTregs, Th17) at 3 months and 6 months post-inoculation; however, a reduction in GI species that correlated with these immune cells populations was observed at 9 months. The association between urinary bacteria and the peripheral immune cells changed throughout the disease pathogenesis as urinary bacteria were negatively correlated with the peripheral iTregs cell population but were positively correlated with the level of Th17 cells. Our results indicate a dysbiosis in the GI and urinary microbiomes of animals with endometriosis that is concomitant with alterations in the levels of peripheral nTregs, iTregs (immune tolerant), and Th17 (inflammatory) cell populations. However, the mechanism(s) of action between specific GI/UG species and host immunity during endometriosis still needs further investigation.

In summary, our findings provide evidence that there may be a unique microbiome “signature” in the GI and UG tracts, as well as a distinct immune profile that is associated with induction of endometriosis. The major findings of this study are the following: (1) the mucosal microbiomes (GI, UG) exhibited a unique profile at pre-inoculation vs. post-inoculation; (2) a systemic inflammatory phenotype via an increase in the ratio of Th17:Treg cells upon the induction of endometriosis; (3) a correlation between inflammation and alteration of microbial communities throughout disease progression. Our results support interaction between the immune system and mucosal microbial dynamics in patients with endometriosis and warrant further investigations to elucidate how these physiological systems impact the pathogenesis of endometriosis.

Limitation of the study: using non-human primate offers a tremendous advantage such as physiologic similarity to humans and reproducibility of experimental results. But the disadvantage of using these animals include the difficulty of availability and relatively high cost. We acknowledged that there were no control animals in the study over a period of 15 months. However, environmental factors such as diet, infections, antibiotics, and genetic background were controlled in the study. Additionally, the pre-inoculation stage was used as the control to be compared to the disease progression over a period of 15 months after inoculation. Thus, the study design establishes an internal baseline for all biological measurement, and therefore reduces animal variability. This design results in a reduction of error and an increase in power with a limited sample size.

## Materials and methods

### Animal housing and health screening

The study was reported in accordance with ARRIVE guidelines from PLOS ONE editorial team. In the study, all 8 non-human primates lived under the same housing conditions, within the same room throughout the duration of the study. All animals received standardized environmental enrichment such as Kong toys, visual stimulation, and auditory enrichment. They all received the same standardized diet from the same provider, but the food was not irradiated. Animals had been on the same diet for at least 60 days prior to the initiation of the study so the dietary influence on microbial dynamics should be well established.

Tuberculosis (TB) testing and fecal float/smear for parasites were screened for all animals. Animals were purchased from a conventional colony that is known to be positive for Papiine herpesvirus 2 and simian T-cell leukemia virus. Since all animals had these infectious reagents when we sampled them as controls, infectious agents should not affect the changes observed following the induction of endometriosis. Routine health screening consists of the semi-annual TB testing and annual physical exam with CBC/chem panel/fecal flotation. Animals that had a change in health status or require medical intervention were then removed from data analysis. Because our animal care facility has a closed colony, we were able to minimize the exposure and transmission of several infectious disease agents.

### Induction of endometriosis and samples process

Endometriosis was experimentally induced in 8 olive baboons as described previously^12^. Briefly, all animals were of reproductive age and confirmed disease free by a laparoscopic viewing of the abdominal cavity prior to inoculation. At the time of inoculation, autologous menstrual endometrium was deposited in the pouch of Douglas, the uterine fundus, the cul de sac, and the ovaries via a pipelle during laparoscopic surgery. A secondary inoculation was performed at the subsequent menses. Disease progression was monitored by laparoscopic visualization at 4 different timepoints over a period of 15 months after inoculation.

All experiments were performed in accordance with relevant guidelines and regulations. All biological samples were collected in the animal care facility at the University of Illinois, Chicago, IL, USA under protocol #17-037. All procedures were approved by the University of Illinois Institutional Animal Care and Use Committee (IACUC) and Michigan State University. Urine and peritoneal fluid samples, fecal and vaginal swab samples, heparinated peripheral blood and eutopic endometrium were collected at pre-inoculation (pre-inoc) and at four post-inoculation timepoints: 3 months, 6 months, 9 months and 15 months. At 15 months post-inoculation, ectopic endometrial tissues were collected prior to the animal being euthanized.

Urine and peritoneal fluid samples (10–50 ml), without preservative, were centrifuged to collect the cellular debris and stored at − 80 °C until DNA extraction was performed. Fecal and vaginal swabs were immediately placed into separate 1 ml sterile Ca^2+^/Mg^2+^ free phosphate-buffered saline (1X PBS) and stored at − 80 °C until DNA extraction was performed. Peripheral blood mononuclear cells (PBMCs) were extracted from heparinized blood collection vials and stored in 1 ml of freezing medium (90% Fetal bovine serum, 10% Dimethyl sulfoxide) in liquid nitrogen until use. All tissues were further processed for RNA extraction for quantitative RT–PCR.

### Analysis of lymphocytes using fluorescence-activated cell sorter

Flow cytometric analysis was performed on all PBMCs to detect nTregs (CD4^+^CD25^+^Foxp3^+^), iTregs (CD4^+^CD25^−^Foxp3^+^) and Th17 (CD4^+^CD25^−^RORγt) cells by utilizing the protocol from Braundmeier et al. 2012. Briefly, approximately 10^5^ to 10^6^ mononuclear cells were stained directly with anti-human FITC-CD4 (L200; 550628, BD Pharmigen), anti-human APC-CD25 (BC96; 17-0259, eBioscience), anti-human PE-Foxp3 (PCH101; 12-4776; eBioscience) and anti-human PE-RORγt (AFKJS-9; 12-6988; eBioscience) antibodies. Lymphocyte cell populations were sorted using a BD Accuri C6 flow cytometer and its respective software (BD Biosciences). Populations were gated on CD4 fluorescent intensity; CD4^+^ subpopulations were identified by CD25^+^, Foxp3^+^ and RORγt^+^ fluorescent intensity. Treg and Th17 cells were compared within each animal.

### Quantitative RT–PCR

Real-time PCR analyses were performed using the following primer/probe sets from Applied Biosystems: Histone 3.3 primers (Forward: GGCGCTCCGTGAAATTAGAC; Reverse: CGCTGGAAGGGAAGTTTGC; Probe: CGCTGGAAGGGAAGTTTGC), Foxp3 (Hs01085834_m1) and RORγt primers (Forward: TGGACCACCCCCTGCTGAGAAGG; Reverse: CTTCAATTTGTGTTCTCATGACT; Probe: GGGAGCCAAGGCCGG).

Real-time PCR amplification and detection were performed in MicroAmp optical 96-well reaction plates using the QuantStudio™ 3 real-time PCR detection system. Relative fold induction of Foxp3 and RORγt were calculated by the ∆Ct method: ∆Ct = Ct_target gene_ – Ct_H3.3 gene_ (presented with 2^−∆Ct^) in eutopic endometrium at all collection time points and ectopic endometrium at 15 months post-inoculation. The difference between Foxp3, RORγt and H3.3 was normalized to controls (pre-inoculation) for each animal. H3.3 was used as an endogenous control gene.

### Microbial community analysis

DNA extraction was performed on fecal specimens, urine pellets, vaginal samples and peritoneal fluid pellets using a MoBio PowerSoil DNA Isolation kit (Qiagen, Carlsbad, CA). After extraction, the DNA stock concentration was measured using a Qubit™ dsDNA BR (Broad-Range) Assay Kit (Q32850; Invitrogen).

#### 16S rRNA gene amplification and sequencing

Bacterial sequencing targeted the V4 region of the 16S rRNA gene (archaeal/bacterial) with a two-step polymerase chain reaction (PCR) approach using the Illumina Nextera XT sequencing protocol. The forward and reverse primer mixture was modified and amplified as previously described, with four variants of 515F and one 806R primer modified for the Illumina MiSeq platform^43^. The thermal cycler conditions for the primary PCR were: 3 min at 95 °C followed by 35 cycles of 95 °C for 30 s, 55 °C for 30 s and 72 °C for 30 s with a 5 min final extension at 72 °C. The PCR products were purified with Agencourt Ampure XP beads (Beckman Coulter, Indianapolis, IN) and each sample was then individually labeled with a unique set of forward and reverse indexes through a second PCR. The secondary index PCR cycle was the same as above but with only 8 cycles, and the resulting product was again purified with Agencourt Ampure XP beads. These DNA amplicons were normalized, pooled to a final loading concentration of 4 pM with 20% PhiX spike-in and sequenced bi-directionally 250 bases using v2 reagents on the MiSeq platform (Illumina, San Diego, CA) at the University of Tennessee Genomics Core.

#### Sequence bioinformatics analysis

Data were quality filtered and processed using QIIME2^44^. First, paired end reads were merged with a Phred quality threshold of Q30; then a quality assessment was performed by specific filtering conditions in accordance with QIIME2 quality control process (Trim and truncate primers: trim-left-forward and reverse = 10, trunc-len-forward and reverse = 250). Exact sequence variants (ESVs) were clustered using the DADA2 algorithm^44^ and aligned to the Greengenes-reference v. 13.8 database for archaea/bacteria. Finally, artifact sequences or host contamination (i.e. mitochondria, chloroplast or eukaryote) were filtered out.

#### Sequencing statistic

A total of 932,143 sequences were obtained after quality filtering and sequence processing. The average number of sequences per sample was 58,957 for fecal samples, 12,099 for urine samples, 43,549 for vaginal samples and 2457 for the peritoneal cavity. Rarefraction curves were set to account for variation in sequencing depth and according to each sample type: fecal sample with 2500; urine samples with 600; vaginal samples 2500; peritoneal samples with 200. At these cut point of sequences per sample, rarefaction curves plateaued indicating sufficient sequencing for the discovery and investigation of the GI/UG and peritoneal cavity microbial communities.

### Statistical analysis

All results in figures and tables are expressed as mean ± SEM, n values in figure legends indicate the number of independent experiments, unless otherwise indicated. Alpha-diversity and evenness were estimated for each sample using Simpson’s evenness measure E, Simpson’s index diversity, and Faith’s phylogenetic diversity metrics (Faith’s PD) calculated in QIIME2. Microbiome alpha-diversity comparisons between the pre-inoculation and all post-inoculation, and the effect of peripheral immune cells with microbiota were assessed by ANOVA (Qimme2R and phyloseq packages). Beta-diversity (diversity between samples) on both weighted and unweighted UniFrac was conducted to compare the dissimilarity between samples via QIIME2. A constrained analysis of principal coordinates ([CAP], capscale function in vegan package) was calculated for bacteria in GI/UG, peritoneal cavity samples with the level of peripheral circulating immune cells included as predictor variables. Variation in community composition among samples was visualized via a non-metric multidimensional scaling plot (NMDS) based on weighted and unweighted Unifrac with phyloseq package. Statistical differences in community composition were assessed using PERMANOVA in QIIME2 with 999 permutations to measure factors driving bacterial community composition^45,46^. For taxon abundance, raw counts were retained and normalized by clr transformation; one-way ANOVA was used to study how presence of disease influenced taxon abundances. Pearson correlations were performed using QIIME to assess the relationships between the GI/UG diversity and level of immune cells in peripheral blood samples.

Non-parametric tests were used to determine differences between study time points when the data set was not normally distributed. Mann–Whitney *U* test was used to determine differences in immune populations for both peripheral blood and endometrial tissue analyses. A value of *P* < 0.05 was considered statistically significant. Data analysis was conducted using GraphPad Prism 7.

## Supplementary Information

Supplementary Legends.

Supplementary Figure S1.

Supplementary Figure S2.

Supplementary Table S1.

Supplementary Table S2.

Supplementary Table S3.
