# Supplementary material for: Effects of endometriosis on immunity and mucosal microbial community dynamics in female olive baboons
Source: Sci Rep. 2022 Jan 31;12:1590. doi: 10.1038/s41598-022-05499-y (PMC8803974; doi:10.1038/s41598-022-05499-y)
Supplement: Supplementary file 1 — Supplementary Legends. [file 41598_2022_5499_MOESM1_ESM.docx]

**Supplementary Figure S1.** Non-metric dimensional scaling ordination of beta diversity analysis using UniFrac for vaginal and peritoneal microbial composition for 8 non-human primates. **A.** Vaginal samples. **B.** Peritoneal fluid samples.

**Supplementary Figure S2.** Level 2 (phyla) taxonomical summary plots for vaginal specimens and peritoneal fluid samples of 8 non-human primates from pre- and post-inoculation of endometriosis. Samples were collected from animal at the pre-inoculation (pre-Inoc) (left panel) and following disease induction (right panel). **A.** Vaginal samples. **B.** Peritoneal fluid samples.
