# Supplementary figures and images for: Effects of endometriosis on immunity and mucosal microbial community dynamics in female olive baboons
Source: Sci Rep. 2022 Jan 31;12:1590. doi: 10.1038/s41598-022-05499-y (PMC8803974; doi:10.1038/s41598-022-05499-y)

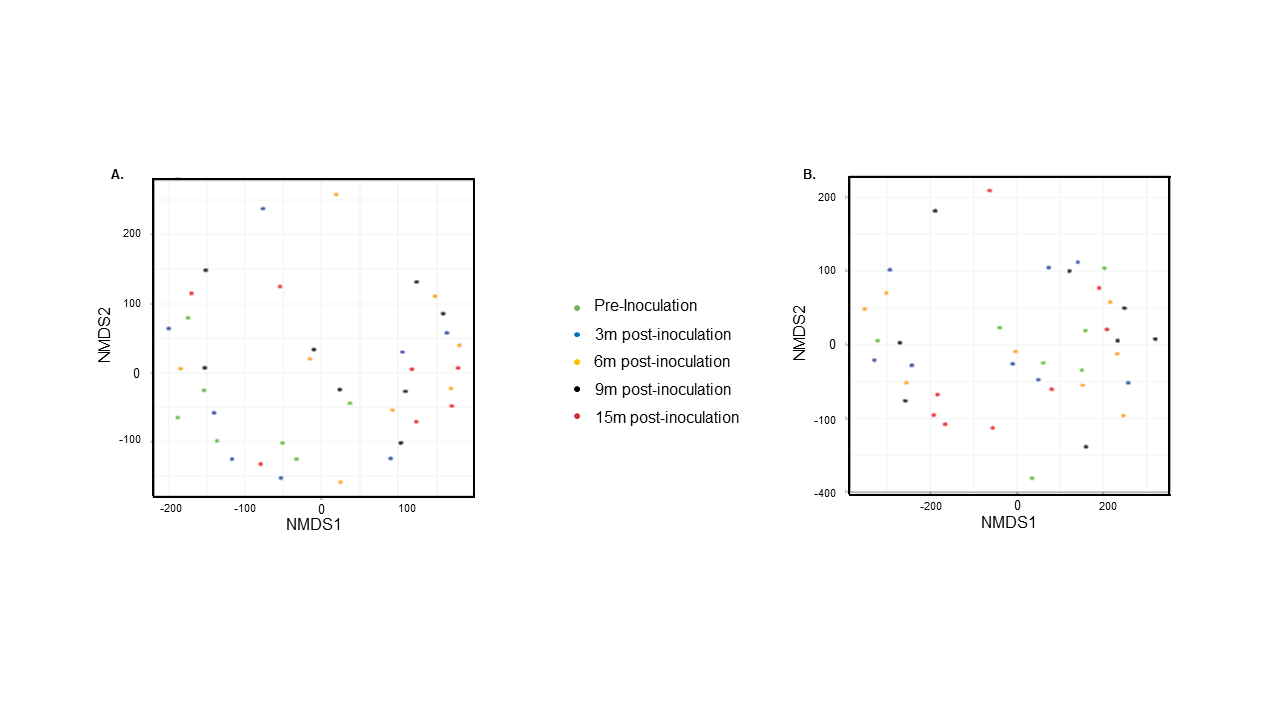

Supplement: Supplementary file 2 — Supplementary Figure S1. [file 41598_2022_5499_MOESM2_ESM.tif]

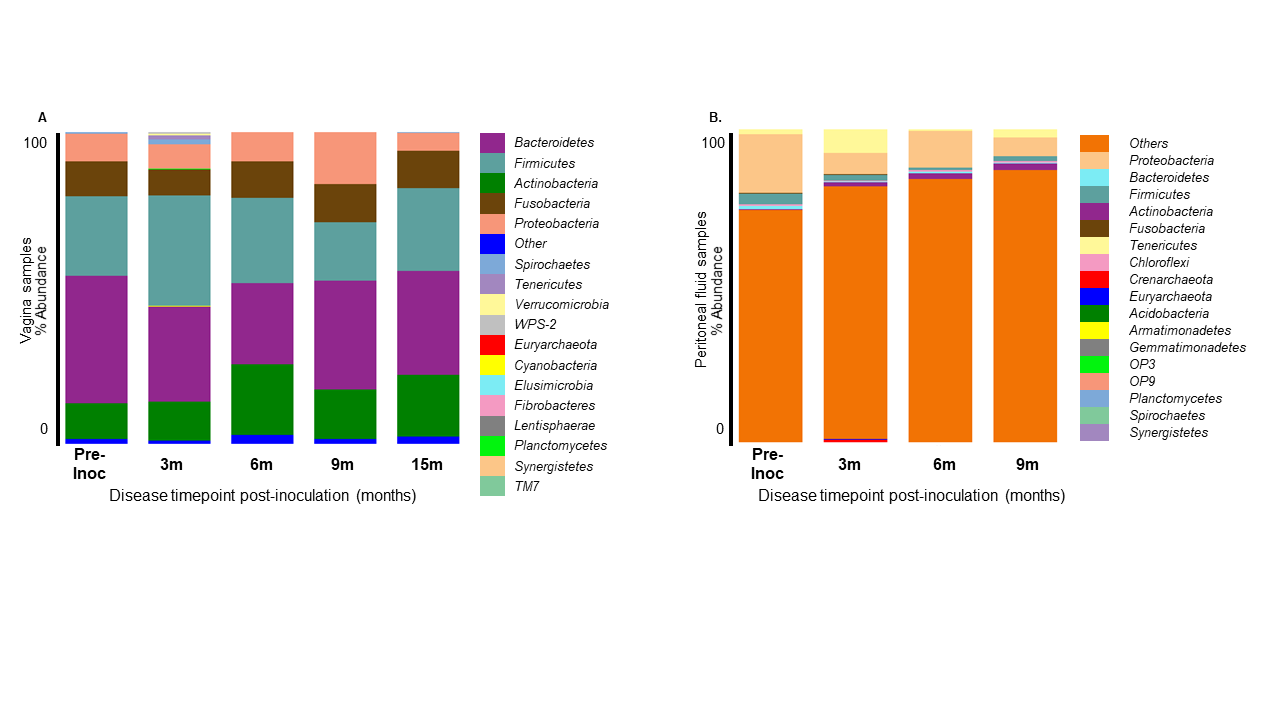

Supplement: Supplementary file 3 — Supplementary Figure S2. [file 41598_2022_5499_MOESM3_ESM.tif]

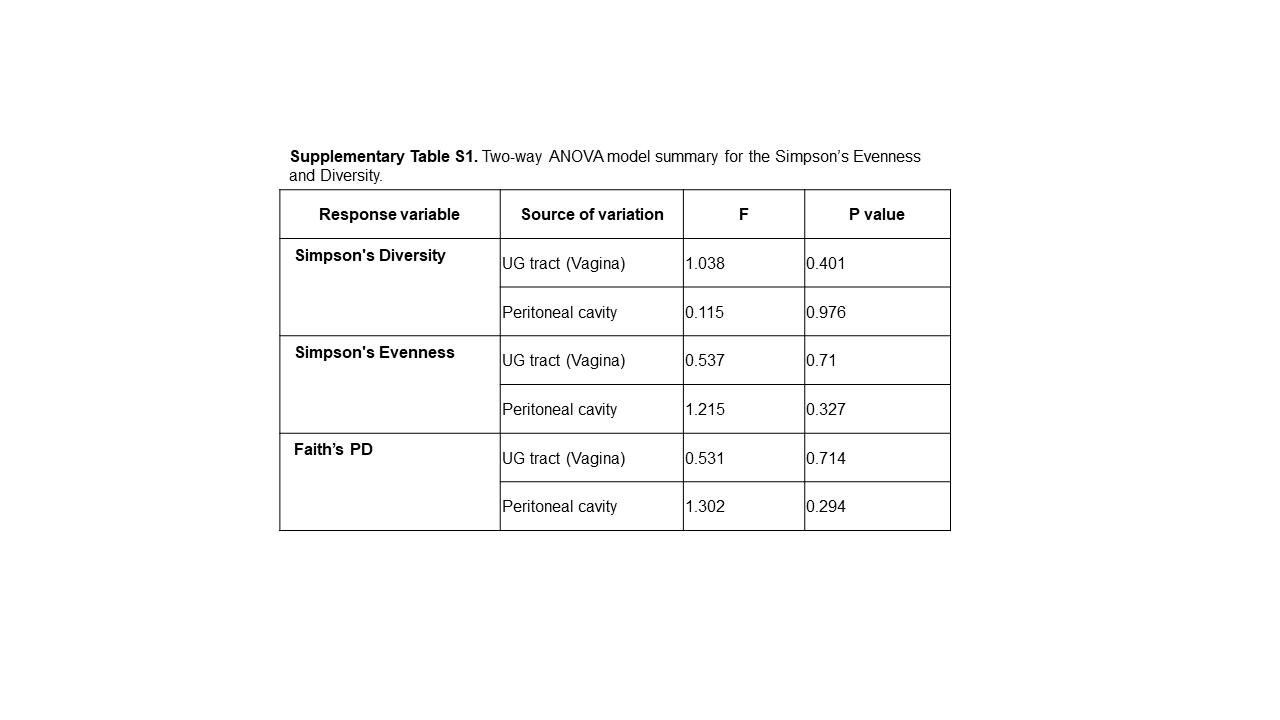

Supplement: Supplementary file 4 — Supplementary Table S1. [file 41598_2022_5499_MOESM4_ESM.tif]

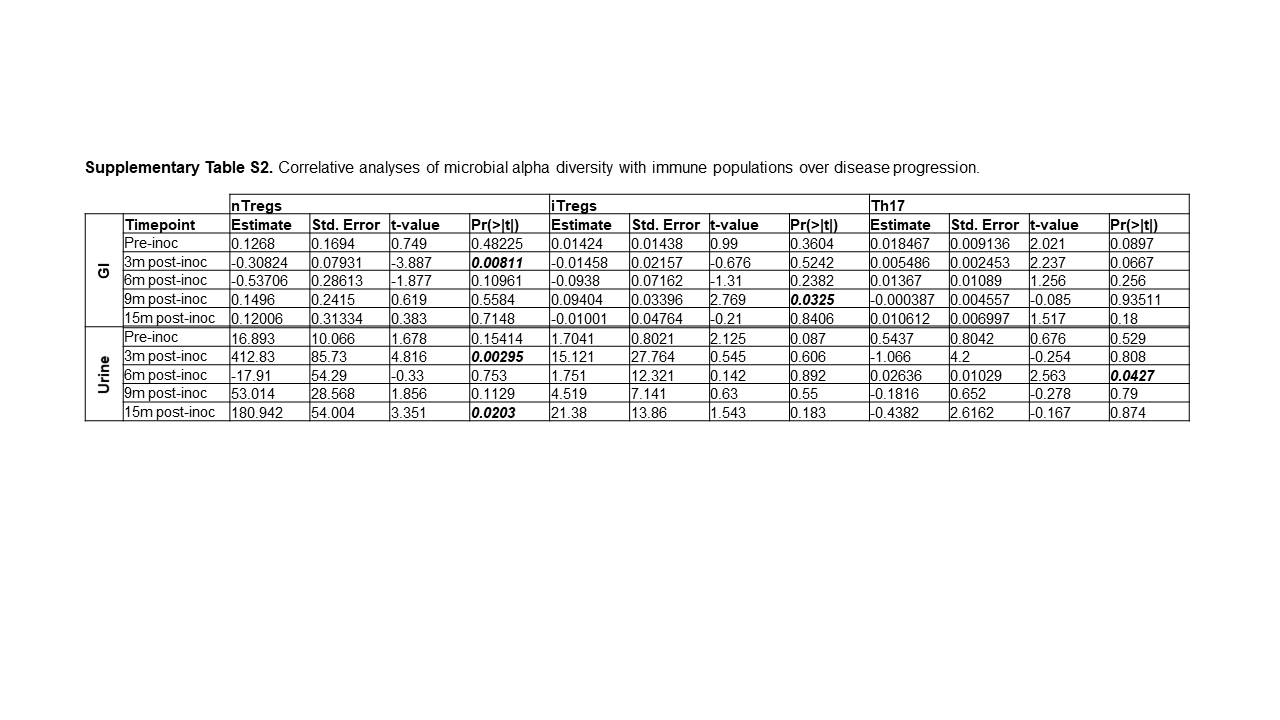

Supplement: Supplementary file 5 — Supplementary Table S2. [file 41598_2022_5499_MOESM5_ESM.tif]

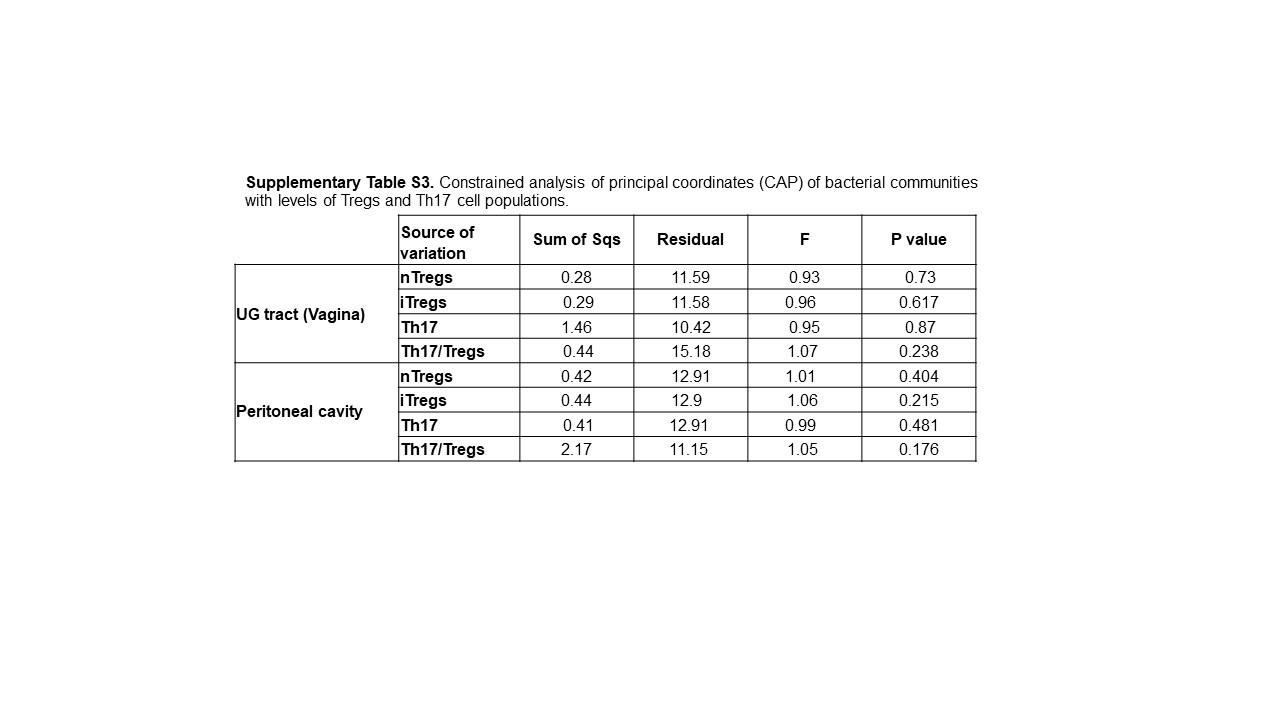

Supplement: Supplementary file 6 — Supplementary Table S3. [file 41598_2022_5499_MOESM6_ESM.tif]
